# Inhibition of receptor activity–modifying protein 1 suppresses the development of endometriosis and the formation of blood and lymphatic vessels

**DOI:** 10.1111/jcmm.15823

**Published:** 2020-09-01

**Authors:** Masako Honda, Yoshiya Ito, Kyoko Hattori, Kanako Hosono, Kazuki Sekiguchi, Kazutake Tsujikawa, Nobuya Unno, Masataka Majima

**Affiliations:** ^1^ Department of Pharmacology Kitasato University School of Medicine Sagamihara Japan; ^2^ Department of Molecular Pharmacology Graduate School of Medical Sciences Sagamihara Japan; ^3^ Department of Obstetrics and Gynecology Graduate School of Medical Sciences Sagamihara Japan; ^4^ Laboratory of Molecular and Cellular Physiology, Graduate School of Pharmaceutical Sciences Osaka University Osaka Japan; ^5^ Department of Medical Therapeutics Kanagawa Institute of Technology Atsugi Japan

**Keywords:** angiogenesis, CGRP, endometriosis, lymphangiogenesis, RAMP1

## Abstract

Neuroimmune interactions are involved in the development of endometriosis. Here, we examined the role of a neuropeptide, calcitonin gene–related peptide (CGRP), and its receptor, receptor activity–modifying protein (RAMP) 1, in growth of endometrial tissues and the formation of blood and lymphatic vessels in a mouse ectopic endometrial transplantation model. Endometrial fragments from donor wild‐type (WT) mice transplanted into the peritoneal wall of recipient WT mice grew with increased density of blood and lymphatic vessels. When tissues from RAMP1‐deficient (RAMP1^−/−^) mice were transplanted into RAMP1^−/−^ mice, implant growth and angiogenesis/lymphangiogenesis were decreased. CGRP was up‐regulated in dorsal root ganglia, and CGRP^+^ nerve fibres were distributed into the implants from the peritoneum. RAMP1 was co‐expressed with CD11b (macrophages) and S100A4 (fibroblasts), but did not co‐localize with blood vessel endothelial cell marker CD31 or lymphatic vessel endothelial hyaluronan receptor (LYVE)‐1. Cultured with CGRP, macrophages up‐regulated vascular endothelial growth factor (VEGF)‐A, VEGF‐C and VEGF‐D, whereas fibroblasts up‐regulated VEGF‐C, but not VEGF‐A or VEGF‐D, in a RAMP1‐dependent manner. CGRP receptor antagonist CGRP8‐37 inhibited growth of and angiogenesis/lymphangiogenesis within endometrial tissue implants. These results suggest that RAMP1 signalling is crucial for growth and angiogenesis/lymphangiogenesis in endometrial tissue. Blockade of RAMP1 is a potential tool for the treatment of endometriosis.

## 1 INTRODUCTION

Endometriosis, characterized by growth of endometrial tissue outside the uterine cavity, is a common gynaecological disease in women of reproductive age.^1^, ^2^ The common symptoms are dysmenorrhoea and infertility, both of which diminish the quality of life.^3^, ^4^ Patients with endometriosis display infiltration of nerve fibres into the endometriotic lesions, and these underly the pain associated with endometriosis.^5^, ^6^ The infiltrated nerves in human peritoneal endometriotic lesions contain nerve fibres expressing calcitonin gene–related peptide (CGRP),^7^ which is recognized as a contributor to chronic pain such as migraine.^8^, ^9^, ^10^ In addition, CGRP^+^ nerve fibres are present in the rat ectopic endometrial transplantation model.^11^

Endometriosis is highly dependent on angiogenesis,^12^ the formation of new blood vessels from pre‐existing blood vessels. Angiogenesis plays a critical role in the growth of ectopic endometriotic lesions, and the maintenance of blood supply is responsible for its survival.^12^, ^13^, ^14^, ^15^ The potent pro‐angiogenic cytokine vascular endothelial growth factor (VEGF)‐A is markedly expressed in peritoneal endometriosis lesions and is associated with an increase in endothelial cells therein.^12^ It has been suggested that endometriosis in humans correlates with enhanced expression of pro‐lymphangiogenic cytokines and lymphatic endothelial cell markers.^12^ Although the evidence suggests that the growth of new lymphatic vessels is associated with endometriosis, the contribution of lymphangiogenesis to the progression of endometriosis is not well understood.

CGRP exerts its action through the CGRP receptor, a heterodimer comprising three subunits: the single membrane‐spanning receptor activity–modifying protein (RAMP) 1, which affords specificity for CGRP binding; a seven‐transmembrane G protein calcitonin receptor‐like receptor (CLR); and receptor component protein.^10^, ^16^ Accumulated evidence indicates that the nervous system closely interacts with the immune system. For instance, the neuropeptide CGRP regulates immune functions,^10^, ^17^ acts on several kinds of immune cells, such as dendritic cells^18^ and macrophages,^19^, ^20^ and down‐regulates inflammation by inhibiting pro‐inflammatory cytokines through RAMP1 signalling in immune cells.^18^, ^19^, ^20^ Furthermore, we reported that the CGRP/RAMP1 axis in the nervous system regulates angiogenesis in tumour tissues^21^ and in injured tissues caused by gastric ulcers^22^ and ischaemia.^23^ CGRP/RAMP1 also plays a role in lymphangiogenesis in mice with skin wounds^24^ and with inflammation in the tail^25^ and diaphragm.^26^ In these pathological conditions, CGRP/RAMP1 signalling facilitates angiogenesis and lymphangiogenesis by inducing pro‐angiogenic/lymphangiogenic growth factor production by accumulated immune cells and fibroblasts.

However, the role of RAMP1 signalling in the regulation of angiogenesis/lymphangiogenesis and in the development of endometriosis remains unknown. In this study, we examined whether endogenous CGRP is involved in the growth of ectopic endometriosis and blood and lymphatic vessels. We found that RAMP1 signalling in macrophages and fibroblasts plays a role in growth and angiogenesis/lymphangiogenesis in endometrial tissue. Our results suggest that blocking RAMP1 may be a promising option for the treatment of endometriosis.

## 2 MATERIALS AND METHODS

### 2.1 Animals

Female RAMP1‐deficient (RAMP1^−/−^) mice (8‐week‐old) were generated as described previously.^18^ Female C57BL/6 WT mice (8‐week‐old) were purchased from CLEA Japan. Mice were maintained in a facility with constant humidity (50% ± 5%) and temperature (25°C ± 1°C) on a 12‐hour light/dark cycle and were provided with food and water ad libitum. All experimental procedures were approved by the Animal Experimentation and Ethics Committee of the Kitasato University School of Medicine and were performed in accordance with the guidelines for animal experiments set down by the Kitasato University School of Medicine.

### 2.2 Endometrial transplantation model

Endometrial transplantation was performed as described previously.^15^, ^27^ Briefly, mice were anaesthetized by intraperitoneal (i.p.) injection of a mixture of 0.75 mg/kg medetomidine hydrochloride (Nippon Zenyaku Kogyo Co., Ltd.), 4.0 mg/kg midazolam (Astellas Pharma Inc) and 5.0 mg/kg butorphanol (Meiji Seika Pharma Co., Ltd.). Then, through paravertebral incisions, the mice were bilaterally ovariectomized to exclude the effects of endogenous oestrogen and menstrual cycle. After the procedure, the effect of medetomidine was reversed by i.p. injection of 0.75 mg/kg atipamezole (Nippon Zenyaku Kogyo Co., Ltd.), and the animals were allowed to recover. All donor and recipient mice received subcutaneous injections of estradiol dipropionate (100 mg/kg) in sesame oil (Obahormone Depot, Aska) every week from the time of ovariectomy. Seven days after ovariectomy, the uterine horns from the donor were removed. A round endometrial fragment (3 mm in diameter) was transplanted to each side of the peritoneal wall of recipient mice and secured using 7‐0 polypropylene sutures (Ethicon, Johnson & Johnson). The day of implantation was defined as day 0. WT or RAMP1^−/−^ recipient mice bearing an implant from donor WT or RAMP1^−/−^ mice are referred to hereafter using the terms WT → WT and RAMP1^−/−^ → RAMP1^−/−^.

### 2.3 Treatment with CGRP8‐37 antagonist

After the endometrial tissue transplantation procedure, an osmotic pump (Alzet osmotic pump; Muromachi‐Kikai Co.) containing the CGRP antagonist CGRP8‐37 (Peptide Institute, Inc) in physiological saline or vehicle was placed into the subcutaneous tissue of the back of WT → WT mice. Then, CGRP8‐37 or vehicle was continuously infused for 14 days with a delivery rate of 80 μg/d.^19^

### 2.4 Tissue harvesting

After endometrial tissue implant transplantation, the two implanted tissue fragments were removed on the indicated days under anaesthesia as described above. Day 0 samples were collected immediately after the implantation. They were sewn into position and then immediately removed with no skin wound suturing. Digital images of the removed endometrial tissues were collected and from them the area of one face of the implant was calculated using ImageJ software (National Institutes of Health). The results are expressed as the areas of the implants per mm^2^. One implant sample was prepared for real‐time reverse transcriptase‐polymerase chain reaction (RT‐PCR) analysis and the other for histology.

### 2.5 Immunohistochemical analysis

After fixation in 10% formaldehyde, tissues were embedded in paraffin, and 3‐µm‐thick sections were used for immunostaining. The sections were incubated at 4°C overnight with a polyclonal rabbit anti‐CD31 antibody (Abcam) or a polyclonal rabbit anti‐lymphatic vessel endothelial hyaluronan receptor (LYVE)‐1 antibody (Abcam) as described previously.^14^

### 2.6 Immunofluorescence analysis

Fixed samples of endometrial lesions were embedded in Tissue‐Tek OCT Compound (Sakura Finetek USA, Inc) and frozen at −80°C before cutting 8‐μm sections using a cryostat. The sections were incubated overnight with the following primary antibodies at 4°C: rabbit anti‐mouse RAMP1 polyclonal antibody (Santa Cruz Biotechnology), rabbit anti‐mouse CGRP polyclonal antibody (Sigma‐Aldrich), rat anti‐mouse CD31 monoclonal antibody (Abcam), rabbit anti‐LYVE‐1 polyclonal antibody (Abcam), goat anti‐LYVE‐1 polyclonal antibody (R&D Systems Inc), rabbit anti‐mouse VEGF‐A polyclonal antibody (Abcam), rat anti‐mouse VEGF‐A monoclonal antibody (BioLegend), rabbit anti‐VEGF‐C polyclonal antibody (Abcam), mouse anti‐VEGF‐C monoclonal antibody (Santa Cruz Biotechnology), goat anti‐VEGF‐D polyclonal antibody (R&D Systems Inc), rat anti‐mouse CD11b monoclonal antibody (BD Biosciences) or goat anti‐mouse S100A4 polyclonal antibody (Abcam). After washing in PBS, the sections were incubated with a species‐appropriate secondary antibody from the following list for 1 hour at room temperature: Alexa Fluor 488‐conjugated donkey anti‐rabbit IgG, Alexa Fluor 488‐conjugated donkey anti‐rat IgG, Alexa Fluor 488‐conjugated donkey anti‐goat IgG, Alexa Fluor 594‐conjugated donkey anti‐rabbit IgG, Alexa Fluor 594‐conjugated donkey anti‐rat IgG or Alexa Fluor 594‐conjugated donkey anti‐goat IgG (all from Molecular Probes). Stained sections were observed, and images were captured using a fluorescent microscope (Biozero BZ‐9000, ×400 magnification; Keyence). The number of CD11b^+^ or S100A4^+^ cells was determined as the average of four fields within the endometrial tissue.

### 2.7 Determination of vessel density

Microvessel density (MVD) in areas within the endometrial tissue implants was used as a measure of angiogenesis, as previously described.^15^ Briefly, blood vessels were stained with an anti‐CD31 antibody, and individual CD31^+^ blood vessels within the area were counted in a single × 400 field and then repeated for a total of four randomly selected areas. The results are expressed as the number of blood vessels per square millimetre (MVD/mm^2^). Lymphangiogenesis was estimated by calculating lymphatic vessel density (LVD) and lymphatic vessel area (LVA), as described previously.^25^ Briefly, lymphatic vessels were stained with anti‐LYVE‐1 antibody, and the individual lymphatic vessels within the area were counted as for CD31^+^ cells as described above. The results are expressed as the number of vessels per square millimetre (LVD/mm^2^). The area covered by lymphatic vessels was measured using ImageJ software. The results are expressed as percentages of the total area observed (% LVA).

### 2.8 Quantitative real‐time reverse transcriptase‐PCR analysis

Total RNA was extracted from mouse tissues and homogenized in TRIzol Reagent (Life Technologies). Single‐stranded cDNA was generated from 1 μg total RNA by reverse transcription using a ReverTra Ace qPCR RT Kit (TOYOBO Co., Ltd.), according to the manufacturer's instructions. Quantitative PCR was performed using TB Green Premix Ex Taq II (Tli RNaseH Plus; Takara Bio, Inc). The gene‐specific primers used for real‐time RT‐PCR were designed using Primer3 software (http://primer3.sourceforge.net/) based on data from GenBank. The primer sequences are listed in Table S1. Data were normalized to expression of glyceraldehyde‐3‐phosphate dehydrogenase (GAPDH) in each sample.

### 2.9 Cell preparation and culture

Bone marrow (BM) cells were isolated from the femurs and tibias of 8‐week‐old female WT and RAMP1^−/−^ mice. To generate BM‐derived macrophages, BM cells were cultured in six‐well plates (1.0 × 10^6^ cells per well) and maintained in RPMI 1640 medium containing 10% foetal calf serum (FCS) and macrophage colony‐stimulating factor (20 ng/mL; BioLegend). On day 7, cells stimulated with lipopolysaccharide (LPS; 100 ng/mL; Sigma‐Aldrich) were either left untreated or treated with rat CGRP (10 nmol/L; Peptide Institute) in RPMI 1640 medium for 3 hours.

The murine fibroblast cell line L929 (Cell Bank at RIKEN BioResource Center) was suspended in Dulbecco's modified Eagle's medium containing 10% FCS and plated in six‐well culture plates (3 × 10^5^ cells/well). Fibroblasts stimulated with LPS (100 ng/mL; Sigma‐Aldrich) and rat CGRP (10 nmol/L) were either left untreated or treated with CGRP8‐37 (1 μmol/L; Peptide Institute).

The cultured BM‐derived macrophages and the fibroblasts were then harvested and homogenized in TRIzol (Life Technologies), and the mRNA levels were measured by real‐time RT‐PCR.

### 2.10 Dissection of dorsal root ganglia (DRG)

Under anaesthesia by i.p. injection of a mixture of 0.75 mg/kg medetomidine hydrochloride, 4.0 mg/kg midazolam and 5.0 mg/kg butorphanol, the medulla spinalis was removed, and the DRGs of the L_1–5_ vertebrae were isolated.^26^

### 2.11 Statistical analysis

All results are represented as the mean ± standard deviation (SD). All statistical analyses were performed using GraphPad Prism software, version 8 (GraphPad Software). Unpaired two‐tailed Student's *t* tests were used to compare data between two groups. One‐way analyses of variance, followed by Tukey's post hoc test, were used to compare data from multiple groups. A *P*‐value < .05 was considered statistically significant.

## 3 RESULTS

### 3.1 Endometrial growth is associated with new formation of blood and lymphatic vessels

The area of endometrial tissue implants from WT → WT mice was measured weekly after transplantation (Figure S1A). Over the course of the 21‐day study, the area increased and reached maximal levels on day 14, which was consistent with our previous results.^15^ To identify the blood and lymphatic vessels, the implant tissue was stained by immunofluorescence using antibodies against CD31 and/or LYVE‐1 (Figure S1B). Double immunofluorescence staining for CD31 and LYVE‐1 in whole‐mount implant tissue revealed CD31^+^ blood vessels and LYVE‐1^+^ lymphatic vessels. Subsequently, we counted the numbers of CD31^+^ and LYVE‐1^+^ cells using immunohistochemistry, as this allowed us to more easily identify the area of evaluation of angiogenesis/lymphangiogenesis, and to distinguish the vessels from other stromal tissues and endometrial glands (Figure S1B). Angiogenesis as indicated by MVD had significantly increased by day 7, and the level was maintained to day 21 (Figure S1C). Lymphangiogenesis as indicated by LVD was enhanced on days 14 and 21 (Figure S1D). In addition, lymphatic vessels on day 14 appeared to be large and irregular. These results indicate that the growth of endometrial tissue correlates with angiogenesis and lymphangiogenesis. That MVD was greater than LVD and MVD increased earlier than LVD during the growth of implants from WT → WT mice indicate that angiogenesis proceeded to lymphangiogenesis during the development of the endometrial implant tissues. To examine the involvement of RAMP1 signalling, we measured RAMP1 expression in the implants. The level of mRNA encoding RAMP1 increased and peaked on day 14. The same was true for the mRNA levels of CLR, a component of CGRP receptor (Figure S1E).

### 3.2 Role of RAMP1 in the growth of endometrial implant tissue and expression of RAMP1

**Figure 1:**
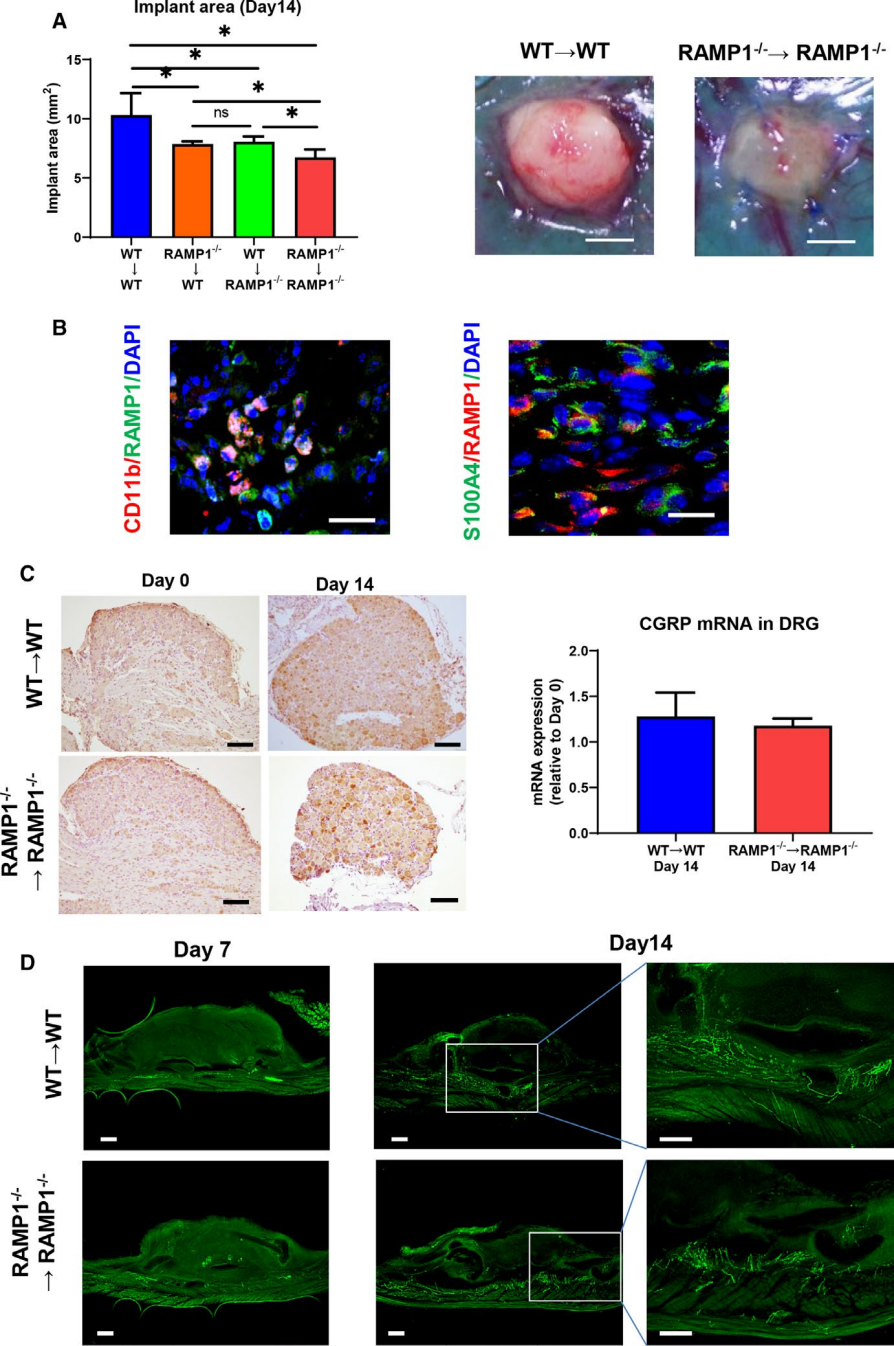
CGRP/RAMP1 signalling is involved in the growth of transplanted endometrial tissue. A, Area of the implants on day 14 after cross‐transplantation of endometrial tissue from WT or RAMP1^−/−^ mice into WT or RAMP1^−/−^ mice. Data are expressed as the mean ± SD (n = 6 mice per group). *P < .05. Right‐hand panels show the typical appearance of implants in the WT → WT and RAMP1^−/−^ → RAMP1^−/−^ mice on day 14 after implantation. Scale bars, 2 mm. B, Double immunofluorescence staining of CD11b (red)/RAMP1 (green) and S100A4 (green)/RAMP1 (red) in implant tissue from WT → WT mice on day 14. Scale bars, 50 μm. C, Immunohistochemical staining of CGRP in lumbar DRG from WT → WT and RAMP1^−/−^ → RAMP1^−/−^ mice on days 0 and 14 after implantation. Scale bars, 100 μm. (Right panel) The level of mRNA encoding CGRP in the lumbar DRG from the WT → WT and RAMP1^−/−^ → RAMP1^−/−^ mice on day 14. Data are expressed as the mean ± SD (n = 4‐6 mice per group). D, Immunofluorescence staining of CGRP (green) in whole‐mount endometrial tissues from WT → WT and RAMP1^−/−^ → RAMP1^−/−^ mice on days 7 and 14. Scale bars, 200 μm

To evaluate the role of RAMP1 signalling in the growth of endometrial implants, we implanted WT or RAMP1^−/−^ endometrial tissues into either WT or RAMP1^−/−^ recipients (Figure 1). When we implanted RAMP1^−/−^ endometrial fragments into WT mice (RAMP1^−/−^ → WT mice), the implant growth on day 14 was suppressed in comparison with that in the WT → WT mice. The same degree of suppression in growth of the implants was seen in the WT → RAMP1^−/−^ mice. The greatest degree of growth suppression was observed with RAMP1^−/−^ → RAMP1^−/−^ mice. These results suggest that the growth of transplanted endometrial fragments is accelerated via RAMP1 signalling from both the implant and the recipient. Based on these results, we subsequently focused on growth of implant and of blood and lymphatic vessels only in WT → WT and RAMP1^−/−^ → RAMP1^−/−^ mice. Immunofluorescence analysis of the implanted endometrial tissue from WT → WT mice on day 14 revealed that RAMP1 was mainly expressed in the interstitial tissues of the implants and at the border of the implanted tissue (Figure S2), and double immunofluorescence analysis revealed that RAMP1 expression co‐localized with that of CD11b and S100A4, suggesting that macrophages or fibroblasts, respectively, are the source of RAMP1 in the implants (Figure 1B).

Because CGRP is produced in the DRG of the nervous system, we next determined the expression of CGRP in the DRG of the L_1–5_ vertebrae of WT → WT and RAMP1^−/−^ → RAMP1^−/−^ implant recipient mice. Immunohistochemical staining with anti‐CGRP antibodies revealed that CGRP expression was similarly enhanced in both WT → WT and RAMP1^−/−^ → RAMP1^−/−^ mice relative to that on day 0 (Figure 1C). There was no significant difference in CGRP mRNA levels in the DRG on day 14 between WT → WT and RAMP1^−/−^ → RAMP1^−/−^ mice.

We also examined whether the endometrial implant tissue was innervated by CGRP nerve fibres. CGRP^+^ nerve fibres in the WT → WT mice were demonstrated by immunofluorescence in the peritoneal wall of the host on day 7, and the distribution appeared to be the same as that in the RAMP1^−/−^ → RAMP1^−/−^ mice (Figure 1D). On day 14, CGRP^+^ nerve fibres were extensively distributed in the peritoneal wall where the implant was grafted, and several CGRP^+^ nerve fibres were infiltrated into the implants. CGRP^+^ fibres in the RAMP1^−/−^ → RAMP1^−/−^ mice were distributed to the same extent as those in the WT → WT mice. These results suggest that CGRP in the nervous system is involved in the progression of endometriosis via the RAMP1 signalling pathway.

### 3.3 Angiogenesis in endometrial implants is suppressed in RAMP1^−/−^ → RAMP1^−/−^ mice

**Figure 2:**
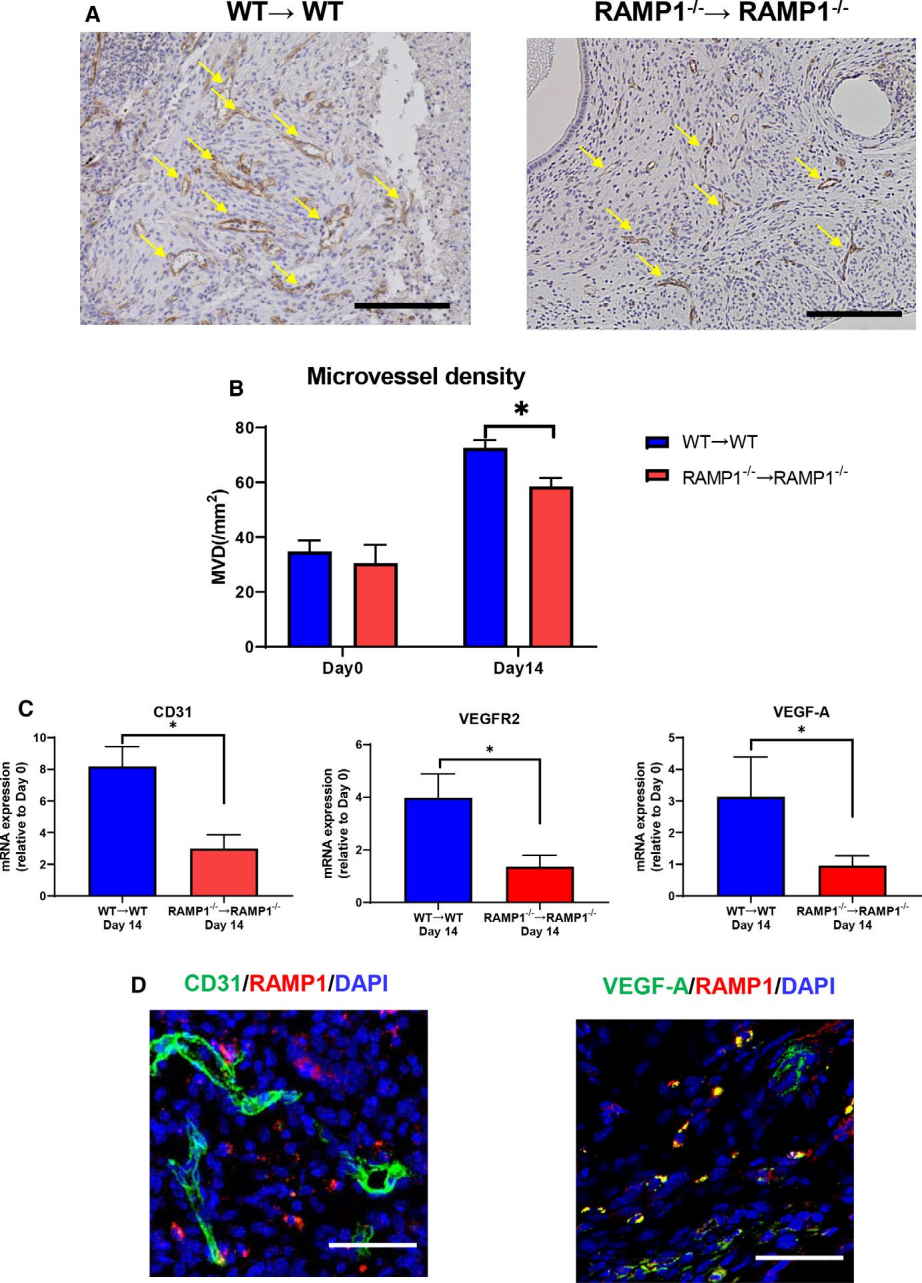
Angiogenesis in the endometrial tissue implants from WT → WT and RAMP1^−/−^ → RAMP1^−/−^ mice. A, Immunohistochemical staining of CD31 in endometrial implant sections from WT → WT and RAMP1^−/−^ → RAMP1^−/−^ mice on day 14. Scale bar, 200 μm. Arrows indicate CD31^+^ cells. B, MVD in the endometrial tissue implants from WT → WT and RAMP1^−/−^ → RAMP1^−/−^ mice on days 0 and 14. Data are expressed as the mean ± SD (n = 4‐5 mice per group). *P < .05. C, The level of mRNA encoding CD31, VEGFR2 and VEGF‐A in endometrial tissue implants from WT → WT and RAMP1^−/−^ → RAMP1^−/−^ mice on day 14. Data are expressed as the mean ± SD (n = 6 mice per group). *P < .05. D, Double immunofluorescence staining of CD31 (green)/RAMP1 (red) and VEGF‐A (green)/RAMP1 (red) in endometrial tissue implants from WT → WT mice on day 14. Scale bars, 50 μm

To elucidate the role of RAMP1 signalling in angiogenesis in endometriosis, we measured the numbers of CD31^+^ blood vessels in the implants of WT → WT and RAMP1^−/−^ → RAMP1^−/−^ mice. There were more newly formed CD31 ^+^ blood vessels in the implants of WT → WT mice than in those of RAMP1^−/−^ → RAMP1^−/−^ mice (Figure 2A). MVD in the implants of WT → WT mice was higher than that in those from the RAMP1^−/−^ → RAMP1^−/−^ mice (Figure 2B). In addition, mRNA expression of CD31 and angiogenesis‐related genes VEGF receptor (VEGFR) 2 and VEGF‐A, in the RAMP1^−/−^ → RAMP1^−/−^ implants were lower than those in the WT → WT implants (Figure 2C).

Double immunofluorescence staining for RAMP1 and CD31 revealed that RAMP1 did not co‐localize with CD31 in the endometrial implant tissues (Figure 2D), indicating that newly formed blood vessels do not express RAMP1. These results suggest that CGRP/RAMP1 signalling does not enhance newly formed blood vessels directly. We found, however, that RAMP1 co‐localized with VEGF‐A expression (Figure 2D), suggesting that the angiogenic response in the endometrial tissue implants was promoted by inducing VEGF‐A via RAMP1 signalling.

### 3.4 Lymphangiogenesis in endometrial implants is suppressed in RAMP1^−/−^ → RAMP1^−/−^ mice

**Figure 3:**
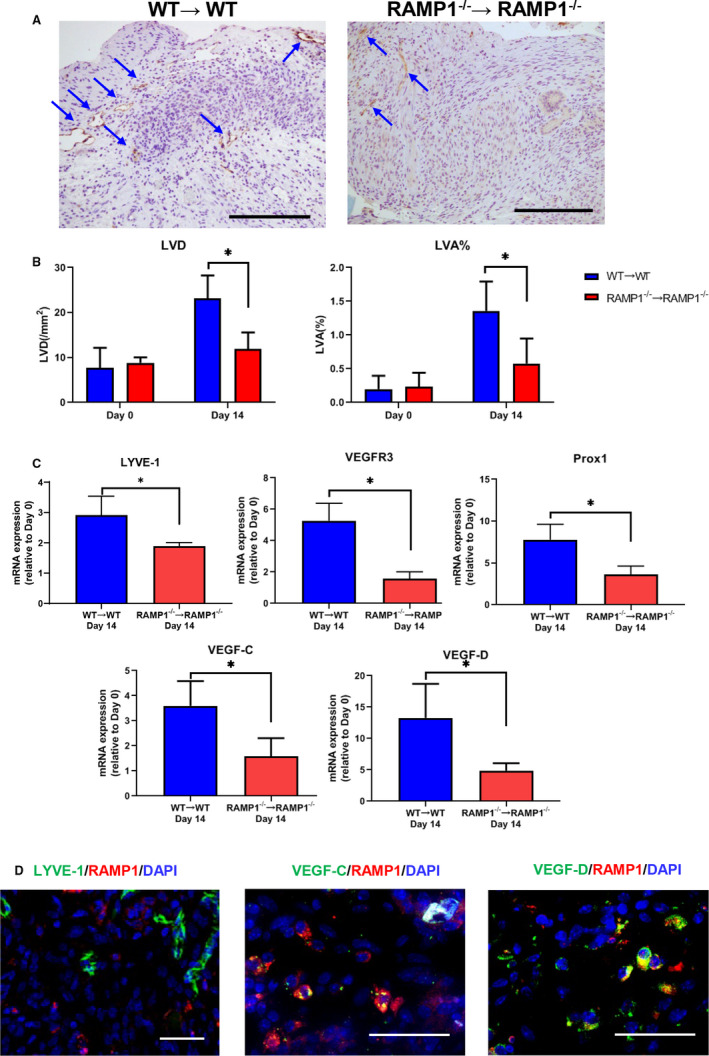
Lymphangiogenesis in the endometrial tissue implants from WT → WT and RAMP1^−/−^ → RAMP1^−/−^ mice. A, Immunohistochemical staining of LYVE‐1 in endometrial implant sections from WT → WT and RAMP1^−/−^ → RAMP1^−/−^ mice on day 14. Scale bars, 200 μm. Arrows indicate LYVE‐1^+^ cells. B, LVD and LVA% in the endometrial tissue implants from WT → WT and RAMP1^−/−^ → RAMP1^−/−^ mice on days 0 and 14. Data are expressed as the mean ± SD (n = 4 mice per group). *P < .05. C, The level of mRNA encoding LYVE‐1, VEGFR3, Prox1, VEGF‐C and VEGF‐D in endometrial tissue implants from WT → WT and RAMP1^−/−^ → RAMP1^−/−^ mice on day 14. Data are expressed as the mean ± SD (n = 5 mice per group). *P < .05. D, Double immunofluorescence staining of LYVE‐1 (green)/RAMP1 (red), VEGF‐C (green)/RAMP1 (red) and VEGF‐D (green)/RAMP1 (red) in endometrial tissue implants from WT → WT mice on day 14. Scale bars, 50 μm

We also examined the role of RAMP1 signalling in lymphangiogenesis as indicated by LVD and LVA% (Figure 3A,B). LVD and LVA% were both higher in the implants of WT → WT mice compared with those of RAMP1^−/−^ → RAMP1^−/−^ mice. Regarding markers of lymphatic endothelial cells, the levels of mRNA encoding LYVE‐1, VEGFR3 and Prospero‐related homeobox 1 (Prox1) in the implants of the WT → WT mice were higher than in those of the RAMP1^−/−^ → RAMP1^−/−^ mice (Figure 3C). Expression of pro‐lymphangiogenic factors, VEGF‐C and VEGF‐D, was also higher in the implants of the WT → WT mice. These results suggest that RAMP1 signalling facilitates lymphangiogenesis by inducing expression of VEGF‐C and VEGF‐D.

Subsequently, we examined localization of RAMP1 expression in lymphatic endothelial cells (Figure 3D). Double immunofluorescence staining for RAMP1 and LYVE‐1 revealed no co‐localization, indicating that newly formed lymphatic vessels do not express RAMP1, and suggesting that RAMP1 signalling does not affect newly formed lymphatic vessels directly. However, RAMP1 expression was found to co‐localize with that of VEGF‐C and VEGF‐D, indicating that RAMP1^+^ cells produce these lymphangiogenic factors.

### 3.5 Recruitment of macrophages and fibroblasts in the implants

**Figure 4:**
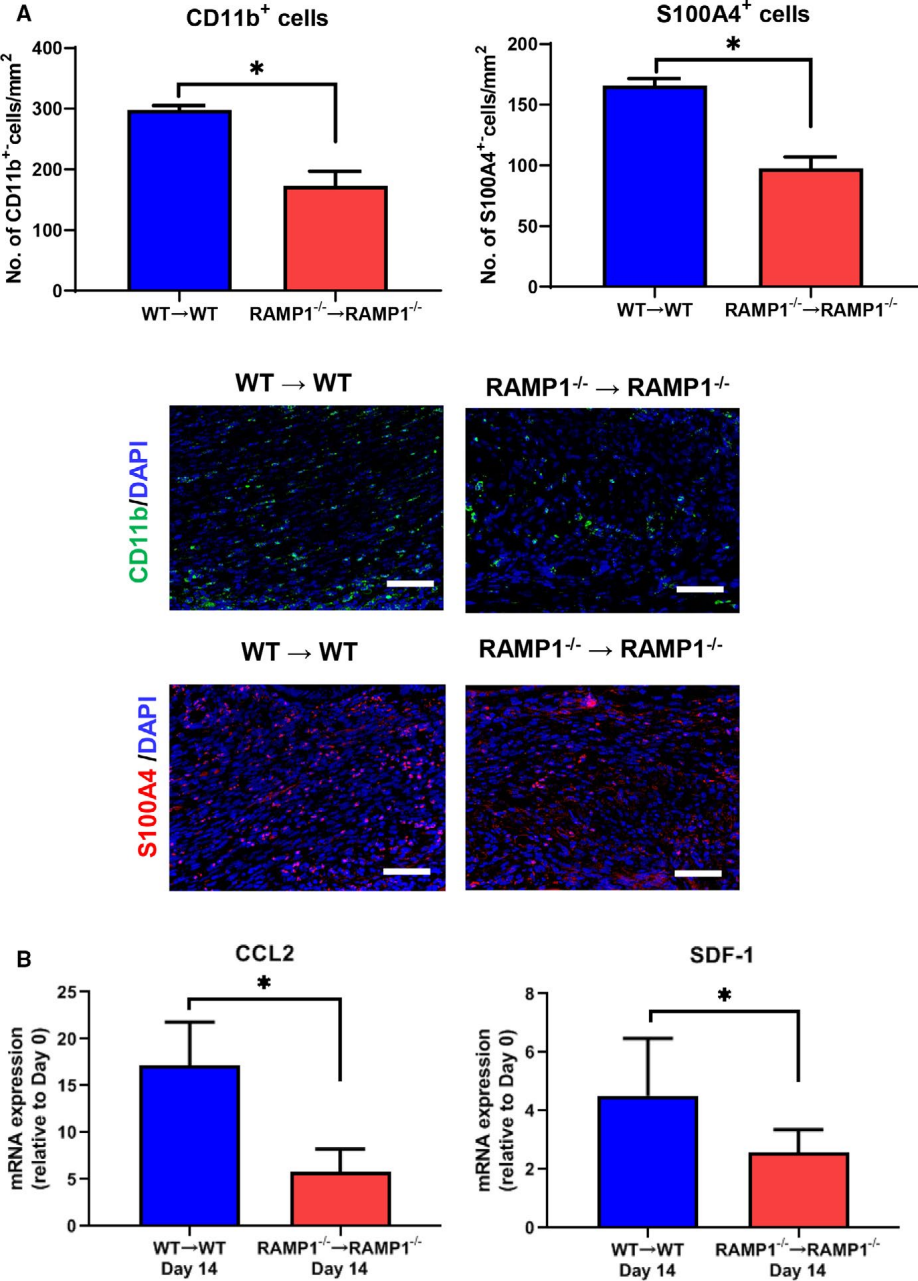
Accumulation of macrophages and fibroblasts in endometrial implant tissues. A, The number of CD11b^+^ cells (macrophages) and S100A4^+^ cells (fibroblasts) in endometrial tissue implants from WT → WT and RAMP1^−/−^ → RAMP1^−/−^ mice on day 14. Data are expressed as the mean ± SD (n = 3‐5 mice per group). *P < .05. Immunofluorescence staining of CD11b (green) or S100A4 (red) in endometrial implant tissue from WT → WT and RAMP1^−/−^ → RAMP1^−/−^ mice on day 14. Scale bars, 50 μm. B, The level of mRNA encoding CCL2 and SDF‐1 in endometrial tissue implants from WT → WT and RAMP1^−/−^ → RAMP1^−/−^ mice on day 14. Data are expressed as the mean ± SD (n = 5‐6 mice per group). *P < .05

We reported that macrophages and fibroblasts are critical players for angiogenesis and lymphangiogenesis during the process of wound healing.^24^, ^28^ We also have shown that macrophages and fibroblasts are key for the growth of endometriosis.^15^ Therefore, we examined the number of CD11b^+^ cells (macrophages) and S100A4^+^ cells (fibroblasts) in the endometrial tissue implants (Figure 4A). The numbers of both CD11b^+^ cells and S100A4^+^ cells were higher in the implants of the WT → WT mice than in those of the RAMP1^−/−^ → RAMP1^−/−^ mice. These results suggest that accumulation of CD11b^+^ cells and S100A4^+^ cells correlates with angiogenesis and lymphangiogenesis in the implants. We also found that enhanced accumulation of CD11b^+^ cells and S100A4^+^ cells in the implants was associated with increased mRNA expression of chemokines, the macrophage chemoattractant C‐C motif chemokine 2 (CCL2) and the fibroblast chemoattractant stromal cell‐derived factor‐1 (SDF‐1; Figure 4B).

### 3.6 Macrophages and fibroblasts express pro‐angiogenic or lymphangiogenic cytokines

**Figure 5:**
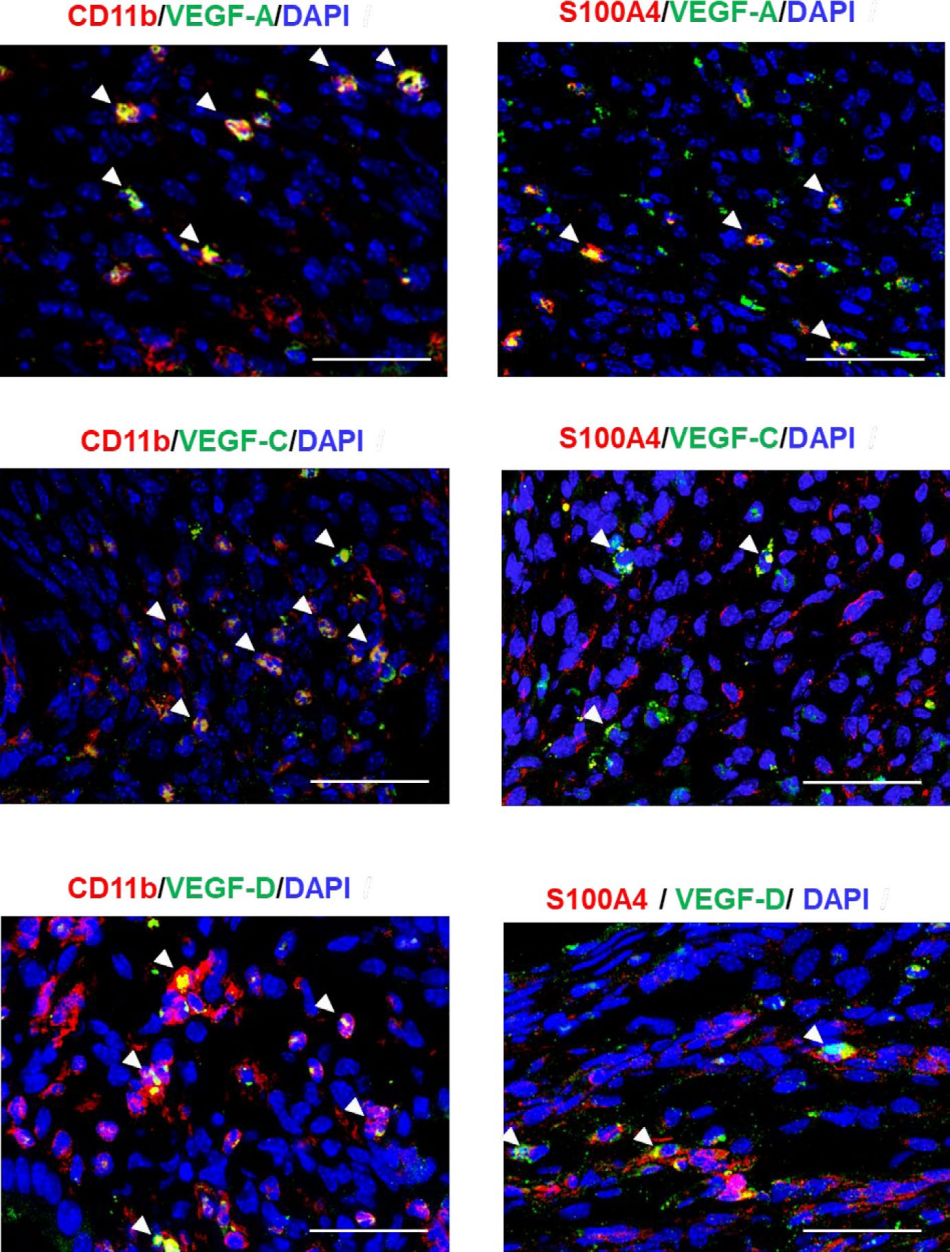
Macrophages and fibroblasts express pro‐angiogenic/lymphangiogenic factors. Double immunofluorescence staining of VEGF‐A (green), VEGF‐C (green) or VEGF‐D (green), with CD11b (red: macrophages) or S100A4 (red: fibroblasts) in endometrial tissue implants from WT → WT mice on day 14. Arrowheads indicate double‐positive cells. Scale bars, 50 μm

Both macrophages and fibroblasts play a critical role in the production of the VEGF family of proteins, members of which are specifically related to the formation of new blood (VEGF‐A) and lymphatic vessels (VEGF‐C and VEGF‐D). We therefore examined whether macrophages and fibroblasts in the implants expressed VEGF‐A, VEGF‐C and VEGF‐D (Figure 5). Immunofluorescence analyses revealed that VEGF‐A expression co‐localized with CD11b^+^ cells and S100A4^+^ cells in the implants from WT → WT mice on day 14, which was consistent with our previous findings.^15^ CD11b^+^ cells and S100A4^+^ cells were also co‐localized with the expression of VEGF‐C and VEGF‐D in the stroma of the implant (Figure 5).

### 3.7 Expression of VEGFs in cultured macrophages and fibroblasts in response to CGRP

**Figure 6:**
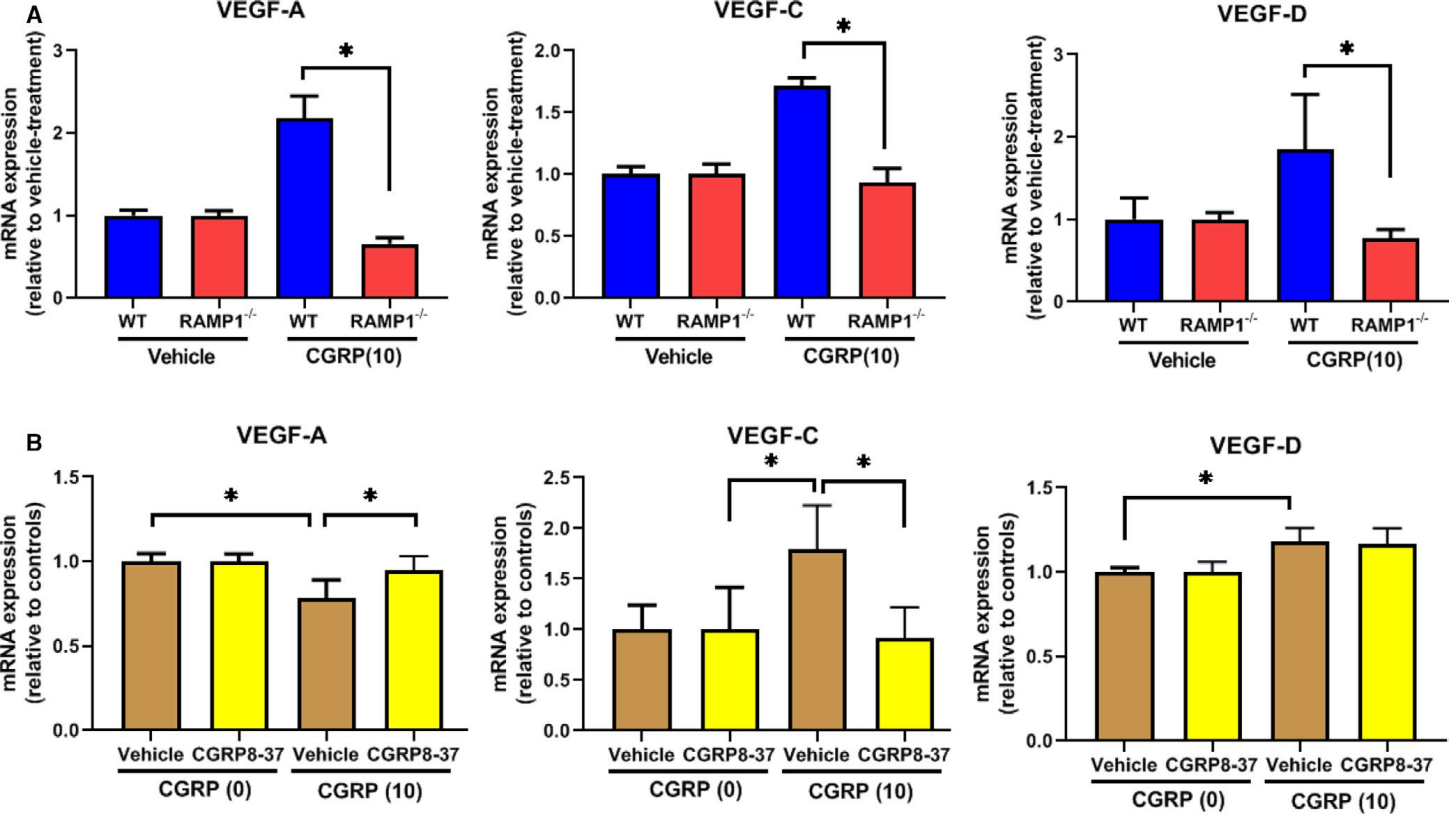
Pro‐angiogenic/lymphangiogenic factors in cultured macrophages and fibroblasts. A, The effects of CGRP (0 and 10 nmol/L) on the level of the mRNA encoding VEGF‐A, VEGF‐C and VEGF‐D in cultured BM‐derived macrophages from WT and RAMP1^−/−^ mice. Data are expressed as the mean ± SD (n = 3‐5 mice per group). *P < .05. B, The effects of CGRP (0 and 10 nmol/L) on the level of the mRNA encoding VEGF‐A, VEGF‐C and VEGF‐D in L929 fibroblasts cultured in the presence of CGRP8‐37 (1 μmol/L) or vehicle. Data are expressed as the mean ± SD (n = 3‐5 mice per group). *P < .05

We examined whether macrophages and fibroblasts express VEGFs in vitro (Figure 6). Cultured WT BM macrophages increased the level of mRNA encoding VEGF‐A, VEGF‐C and VEGF‐D when stimulated with CGRP, whereas similarly treated RAMP1^−/−^ BM macrophages did not (Figure 6A). These results indicate that cultured macrophages up‐regulate VEGF‐A, VEGF‐C and VEGF‐R in a RAMP1‐dependent manner. The level of mRNA encoding VEGF‐A in cultured L929 fibroblasts was slightly down‐regulated by CGRP, which the addition of the CGRP receptor antagonist CGRP8‐37 restored (Figure 6B). However, CGRP stimulation increased the level of mRNA encoding VEGF‐C and VEGF‐D in the cultured fibroblasts, and although CGRP8‐37 decreased the elevated VEGF‐C, it had no effect on the elevated VEGF‐D. These results suggest that fibroblasts increase VEGF‐C but not VEGF‐A or VEGF‐D expression via CGRP receptor signalling.

### 3.8 Inhibition of the CGRP receptor suppresses the growth of implants and new blood and lymphatic vessels

**Figure 7:**
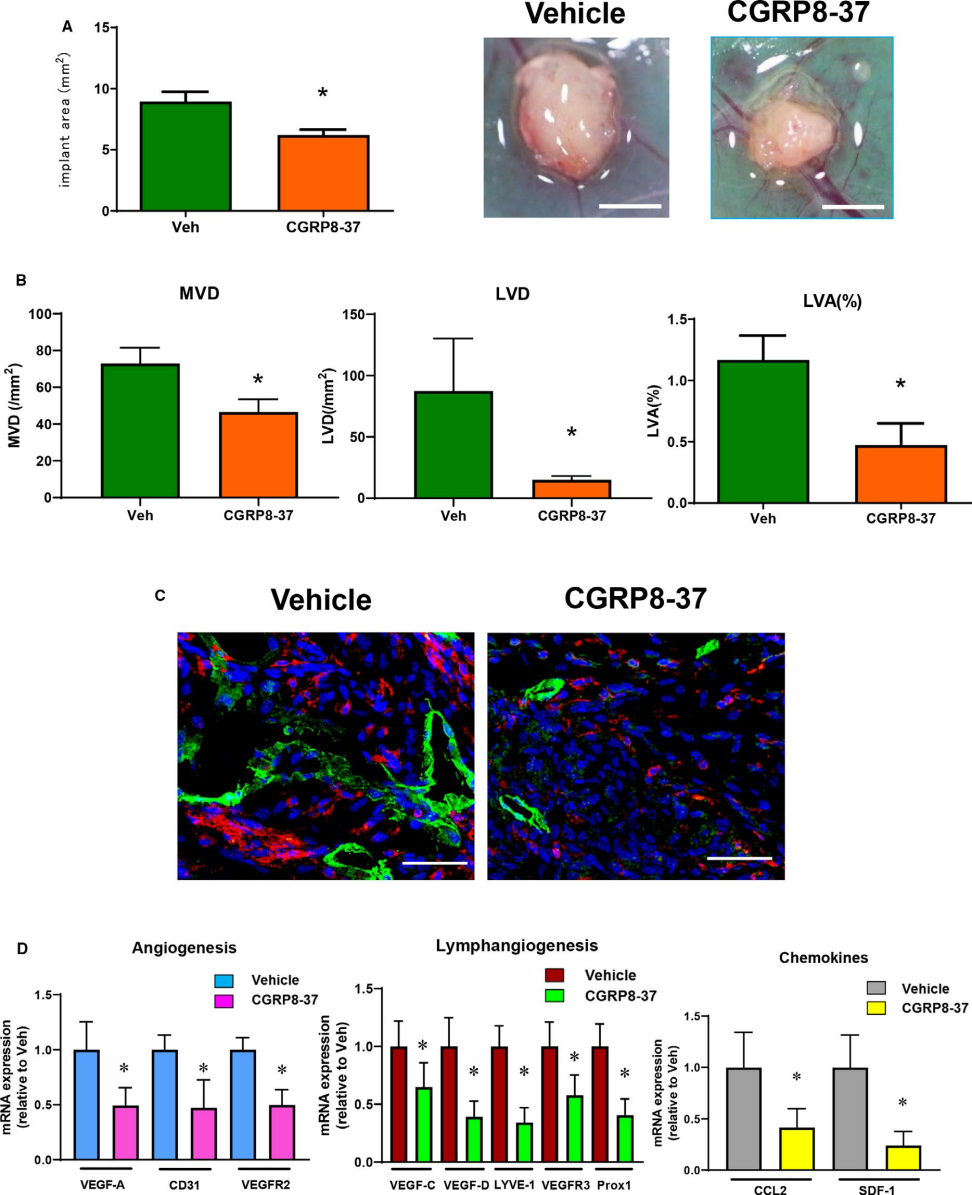
Effect of CGRP receptor antagonist CGRP8‐37 on growth and angiogenesis/lymphangiogenesis in endometrial tissue implants. A, The area of the implants in WT → WT mice treated with continuous administration of CGRP8‐37 or vehicle. Data are expressed as the mean ± SD (n = 6 mice per group). *P < .05. B, MVD, LVD and LVA% in mice treated with continuous administration of CGRP8‐37 or vehicle. Data are expressed as the mean ± SD (n = 4‐6 mice per group). *P < .05. C, Double immunofluorescence staining of CD11b (red) and LYVE‐1 (green) in the endometrial tissue implants from WT → WT mice treated with continuous administration of CGRP8‐37 or vehicle on day 14. Scale bars, 50 μm. D, The level of mRNA expression of pro‐angiogenic genes VEGF‐A, CD31 and VEGFR2; lymphangiogenesis‐related genes VEGF‐C, VEGF‐D, LYVE‐1, VEGFR3 and Prox‐1; and chemokine genes CCL2 and SDF‐1 in mice treated with continuous administration of CGRP8‐37 or vehicle. Data are expressed as the mean ± SD (n = 6 mice per group). *P < .05

Finally, to confirm the role of CGRP/RAMP1 signalling in endometriosis, we examined the effect of CGRP8‐37 on implant growth, angiogenesis and lymphangiogenesis (Figure 7). Treatment of the WT → WT mice with CGRP8‐37 reduced the size of the endometrial tissue implants (Figure 7A). CGRP8‐37 also attenuated angiogenesis in the implant, as indicated by decreased MVD, and also lymphangiogenesis, as indicated by decreased LVD and LVA% (Figure 7B). Further, in the WT → WT mice treated with CGRP8‐37, LVD was more reduced in comparison with LVA%, suggesting that WT → WT mice with CGRP8‐37 had small lymphatic vessels. This was supported by analyses of immunofluorescence staining for LYVE‐1 (Figure 7C). Double immunofluorescence staining for CD11b and LYVE‐1 also revealed no co‐localization of LYVE‐1 expression with CD11b^+^ cells (Figure 7C), indicating that LYVE‐1 was expressed in lymphatic vessels, but not in macrophages in endometrial implant tissue. CGRP8‐37 treatment decreased the level of mRNA encoding CD31, VEGF‐A and VEGFR2 (Figure 7D), and reduced the expression of pro‐lymphangiogenesis genes VEGF‐C, VEGF‐D, VEGFR3, LYVE‐1 and Prox1 in the implants from the WT → WT mice. The expression levels of genes encoding the chemokines CCL2 and SDF‐1 in the implants from WT → WT mice treated with CGRP8‐37 were lower than in those from vehicle‐treated WT → WT mice.

## 4 DISCUSSION

In the present study, we have shown in a mouse ectopic endometriosis model that RAMP1 signalling stimulated the growth of implanted endometrial tissue and was a critical regulator of angiogenesis and lymphangiogenesis therein. Accumulation of RAMP1^+^ macrophages and fibroblasts was shown to be a key driver of growth and angiogenesis/lymphangiogenesis in the endometrial tissue implants. Furthermore, inhibition of CGRP suppressed implant growth and the formation of new blood and lymphatic vessels. The results suggest that blocking RAMP1 signalling will be a promising strategy for the treatment of endometriosis.

Our data indicate that endogenous CGRP contributes to the growth of endometrial tissue implants. That endometriosis is accompanied by pelvic pain implies that the nervous system is deeply involved in its pathogenesis. Endometriotic lesions in humans and rodents are innervated by several kinds of nerve fibres including sensory nerves.^6^ Indeed, CGRP^+^ nerve fibres are distributed to the endometriotic lesions,^7^, ^11^ which is consistent with the findings of our present study. Furthermore, we found that CGRP^+^ nerve fibres were extensively distributed within the peritoneal wall, to which the graft was adhered, and extended from the host peritoneal tissues to the implant tissues in both WT → WT and RAMP1^−/−^ → RAMP1^−/−^ mice. In addition, CGRP expression in the lumbar DRG from the WT → WT mice was similar to that in those from the RAMP1^−/−^ → RAMP1^−/−^ mice. The similarity in CGRP levels in the DRG and in the CGRP^+^ innervation of implants in the WT → WT and RAMP1^−/−^ → RAMP1^−/−^ mice demonstrates that RAMP1 signalling is crucial for the development and maintenance of endometriosis.

Angiogenesis plays important roles not only in physiological menstruation but also in endometriosis.^12^ The current studies demonstrated that RAMP1 signalling is essential for angiogenesis and growth in endometrial tissue implants by increasing levels of the angiogenic growth factor VEGF‐A accompanied by an increase in endothelial cell markers including CD31 and VEGFR2. However, it should be noted that CD31 and VEGFR2 are expressed in endothelial cells of blood vessels as well as in lymphatic vessels. Additionally, RAMP1 signalling facilitated angiogenesis in the implants by inducing VEGF‐A expression but not by the direct action of RAMP1 on angiogenic endothelial cells. We previously reported that angiogenesis in endometrial implants is dependent on VEGF‐A.^14^, ^29^ Using the same endometrial transplantation model, we also showed that signalling through a ligand of VEGF‐A, VEGFR1, is responsible for angiogenesis and growth in implanted endometrial tissue.^15^ The mechanisms by which endometriotic lesions establish remain elusive; however, the formation of new blood vessels in the endometriotic tissues is involved in the maintenance and progression of endometriosis by supplying oxygen and essential nutrients.

Furthermore, angiogenesis in endometrial tissues is associated with infiltration of macrophages. The accumulated macrophages in transplanted endometrial tissue are crucial for angiogenesis and growth.^13^, ^15^ Our present data indicate that RAMP1 signalling plays a role in VEGF‐A generation by the accumulated macrophages in the endometriotic tissue. We found that cultured BM macrophages up‐regulated VEGF‐A expression in a RAMP1 signalling‐dependent manner. Thus, immune cells contribute to angiogenesis and the progression of endometriosis via RAMP1 signalling, indicating that neuroimmune interactions play important roles in the pathogenesis of endometriosis. RAMP1 signalling in immune cells attenuates inflammation in the skin and lung.^30^ We also showed that CGRP interacts with immune cells to regulate inflammatory cytokines, which results in reducing chemical‐induced inflammation in the intestine^19^ and liver.^20^ In addition to its role in regulating inflammation, we showed that CGRP/RAMP1 signalling interacts with macrophages and fibroblasts to enhance angiogenic responses in mice with skin wounds,^24^ peritonitis^26^ and, as demonstrated in the present study, endometriosis. Accumulated fibroblasts in endometrial tissue implants appear to promote angiogenesis by inducing VEGF‐A,^15^, ^31^ although in the present study cultured fibroblasts failed to up‐regulate VEGF‐A in response to CGRP, which is consistent with our previous findings.^24^ Taken together, these results indicate that CGRP/RAMP1 signalling enhances angiogenesis in endometrial tissue implants by inducing VEGF‐A expression in macrophages. It is worth noting that angiogenesis preceded lymphangiogenesis during the development of the experimental endometriosis, suggesting that the new formation of blood vessels in the implants that connect with host tissue is an essential step for the initial engraftment of implants.

Compared with angiogenesis, much less is known about the role of lymphangiogenesis in the development and progression of endometriosis. Our data indicate that RAMP1 signalling contributes to the endometrial tissue implant growth and lymphangiogenesis therein. Because RAMP1 does not co‐localize with lymphatic endothelial cells, lymphangiogenesis in the implants was not induced by an autonomous effect of RAMP1 on lymphatic endothelial cells. Rather, RAMP1 signalling apparently mediates lymphangiogenesis by inducing production of pro‐lymphangiogenic growth factors, including VEGF‐C and VEGF‐D, by macrophages and fibroblasts accumulated into the implants. Previously, we showed that macrophages and fibroblasts are involved in lymphangiogenesis during the healing of wounds.^28^ We also reported that macrophages appear to be responsible for inducing lymphangiogenesis through the production of pro‐lymphangiogenic cytokines, VEGF‐C and VEGF‐D, in a RAMP1‐dependent manner,^24^, ^26^ which is consistent with the results of the present studies. These results suggest that neuroimmune crosstalk regulates not only angiogenesis but also lymphangiogenesis during the progression of endometriosis. In addition, the lymphatic vessels in the implant tissue on day 14 appeared to be large and irregular. These structural alterations were accompanied by the growth of endometriosis. These findings may be reflected by the enhanced drainage functions of lymphatic vessels through newly formed enlarged lymphatic vessels, because the increase in lymphatic drainage of fluids from implant tissues is associated with the growth of endometriosis.^32^

Although inhibition of RAMP1 signalling attenuated angiogenesis and lymphangiogenesis, the inhibitory effects were limited. This indicates that other factors than RAMP1 signalling might be involved in angiogenesis/lymphangiogenesis in the mouse ectopic endometrial transplantation model. Because CLR is not only part of the CGRP receptor, but also part of the adrenomedullin receptor, adrenomedullin might contribute to angiogenesis/lymphangiogenesis.^33^, ^34^

In the present study, we used S100A4 as a fibroblast marker.^24^, ^28^ Although S100A4 is expressed in activated fibroblasts and myofibroblasts,^35^ S100A4 is also reported to be expressed by immune cells including macrophages.^36^ Therefore, identifying the fibroblasts specifically involved in endometriosis will be important for understanding the role of angiogenesis/lymphangiogenesis during the development of endometriosis.

Cross‐transplantation of endometrial tissues isolated from genetically engineered mice is helpful for identification of the cellular sources contributing to endometriosis. Our recent studies showed that macrophages derived from the host and partly from the implant contribute to the development of endometriosis.^15^ By contrast, fibroblasts were mainly derived from the grafts. Our present data indicate that RAMP1 signalling in both implant host tissues contributes to growth and angiogenesis/lymphangiogenesis in endometriosis. Although both accumulated macrophages and fibroblasts in the endometrial tissue implants expressed RAMP1, exactly how and from where these cells are attracted to play a role in endometriosis remains unknown.

CGRP8‐37, an antagonist for the CGRP receptor, is a low‐molecular‐weight compound. We found that the continuous administration of CGRP8‐37 significantly suppressed the growth of endometrial tissues and angiogenesis/lymphangiogenesis therein. Recently, RAMP1 signalling has been clinically targeted by antagonists for the treatment of migraines.^9^ Further studies will be needed to clarify whether blockade of RAMP1 signalling rescues patients with endometriosis from chronic pelvic pain and to investigate whether the CGRP/RAMP1 axis offers new potential therapeutic targets for the treatment of endometriosis. In addition, the potential role for the CGRP/RAMP1 pathway in the development of endometriotic lesions should be reproduced and validated in human endometriotic tissue.

In conclusion, the accumulation of RAMP1^+^ macrophages and fibroblasts was the key driver of growth and angiogenesis/lymphangiogenesis in the endometrial tissue implants in a mouse ectopic endometriosis model. RAMP1 inhibition with antibodies or small molecule inhibitors is likely to be a useful tool for the treatment of endometriosis.

## CONFLICTS OF INTEREST

The authors have no conflicts of interest to disclose.

## AUTHOR CONTRIBUTION

**Masako Honda:** Conceptualization (equal); Data curation (equal); Investigation (equal); Writing‐original draft (equal). **YOSHIYA ITO:** Conceptualization (equal); Investigation (equal); Writing‐original draft (equal); Writing‐review & editing (equal). **Kyoko Hattori:** Investigation (equal); Methodology (equal). **Kanako Hosono:** Data curation (equal); Funding acquisition (equal); Methodology (equal). **Kazuki Sekiguchi:** Funding acquisition (equal); Investigation (equal). **Kazutake Tsujikawa:** Resources (equal). **Nobuya Unno:** Supervision (equal). **Masataka Majima:** Funding acquisition (equal); Supervision (equal); Writing‐review & editing (equal).

## Supporting information

Figure S1

Figure S2

Table S1

Supplementary Material

## Data Availability

The data that support the findings of this study are available from the corresponding author upon reasonable request.
