# Supplementary figures and images for: Inhibition of receptor activity–modifying protein 1 suppresses the development of endometriosis and the formation of blood and lymphatic vessels
Source: J Cell Mol Med. 2020 Sep 1;24(20):11984–97. doi: 10.1111/jcmm.15823 (PMC7578853; doi:10.1111/jcmm.15823)

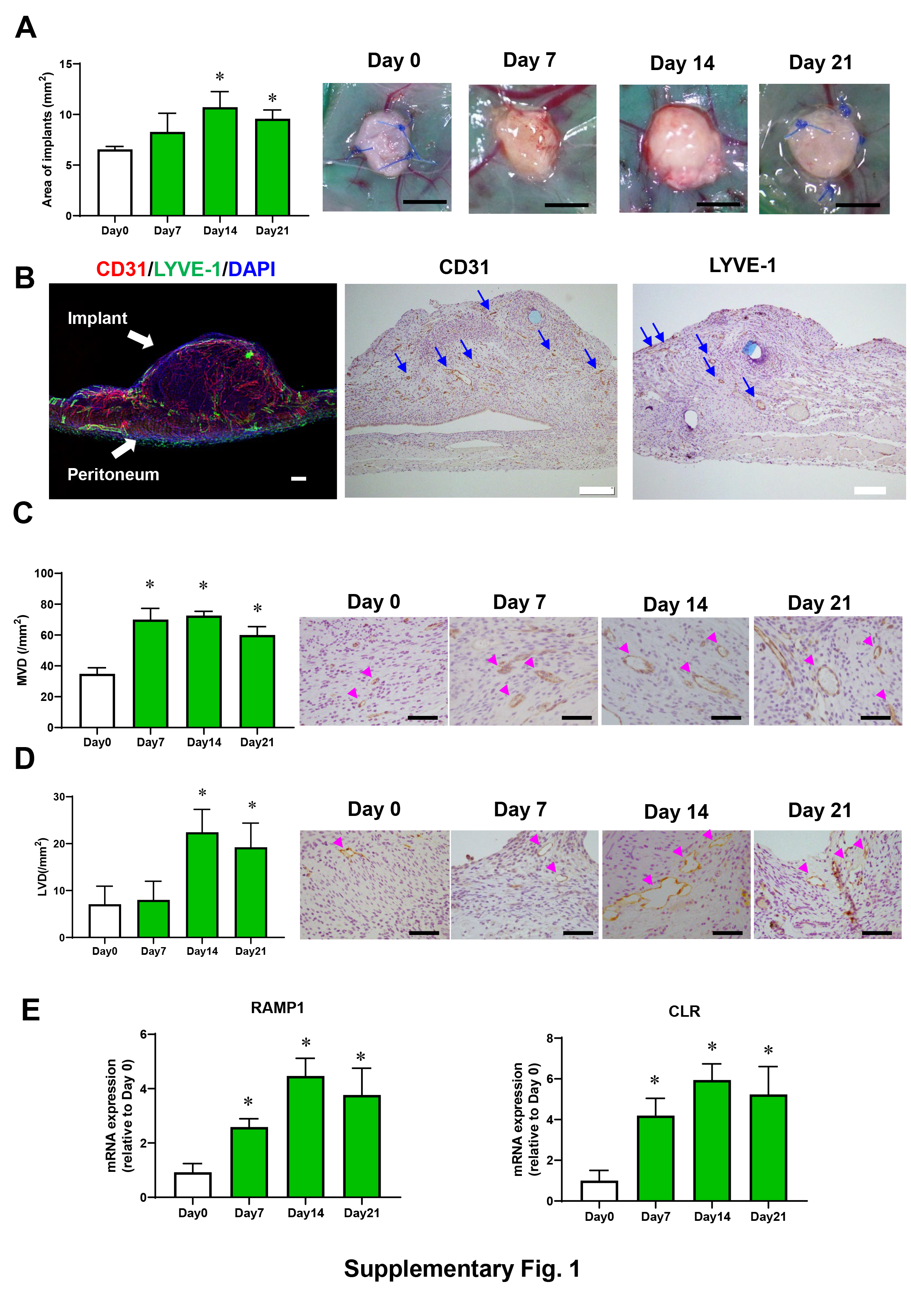

Supplement: Supplementary file 1 — Figure S1 [file JCMM-24-11984-s001.tif]

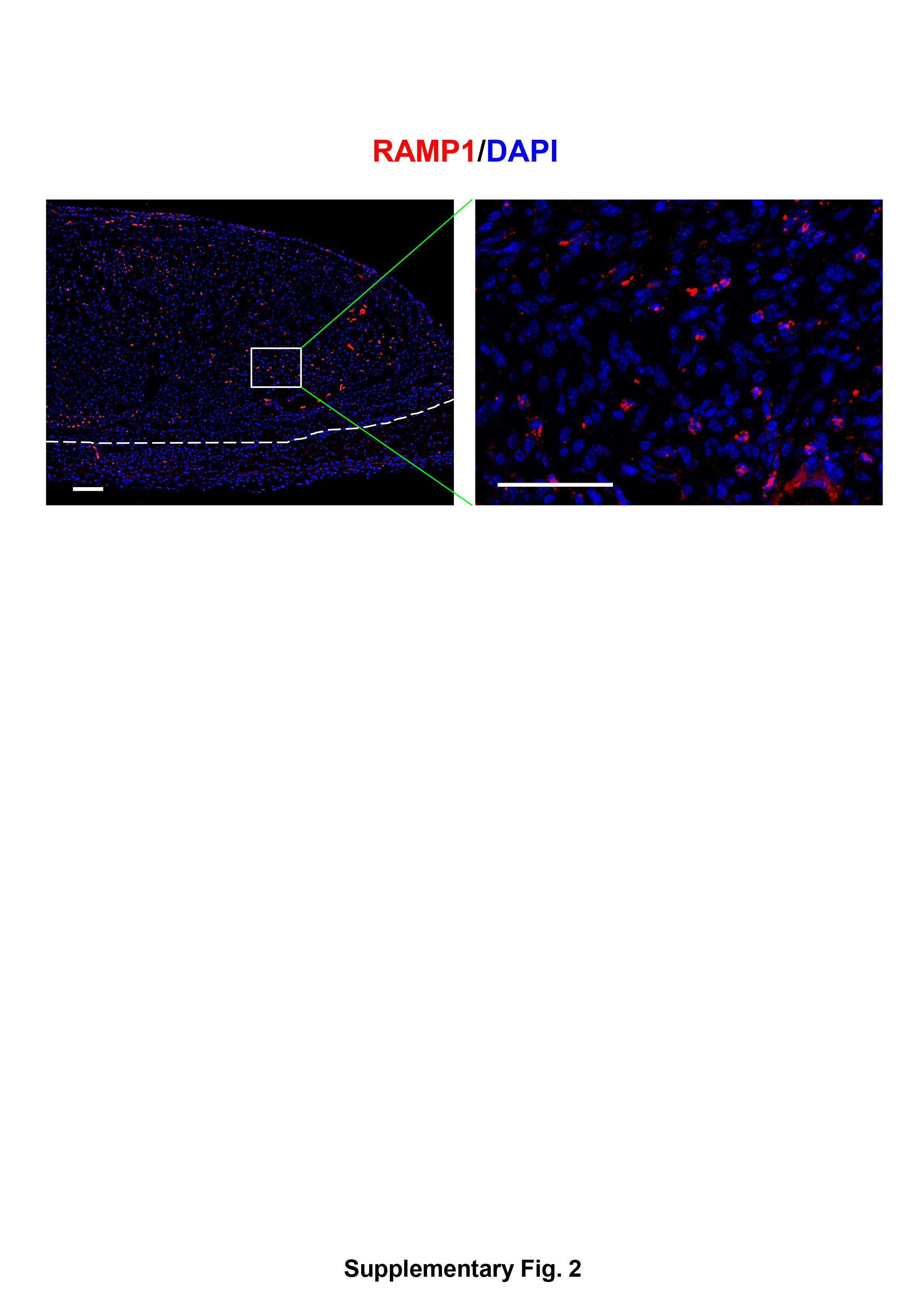

Supplement: Supplementary file 2 — Figure S2 [file JCMM-24-11984-s002.tif]

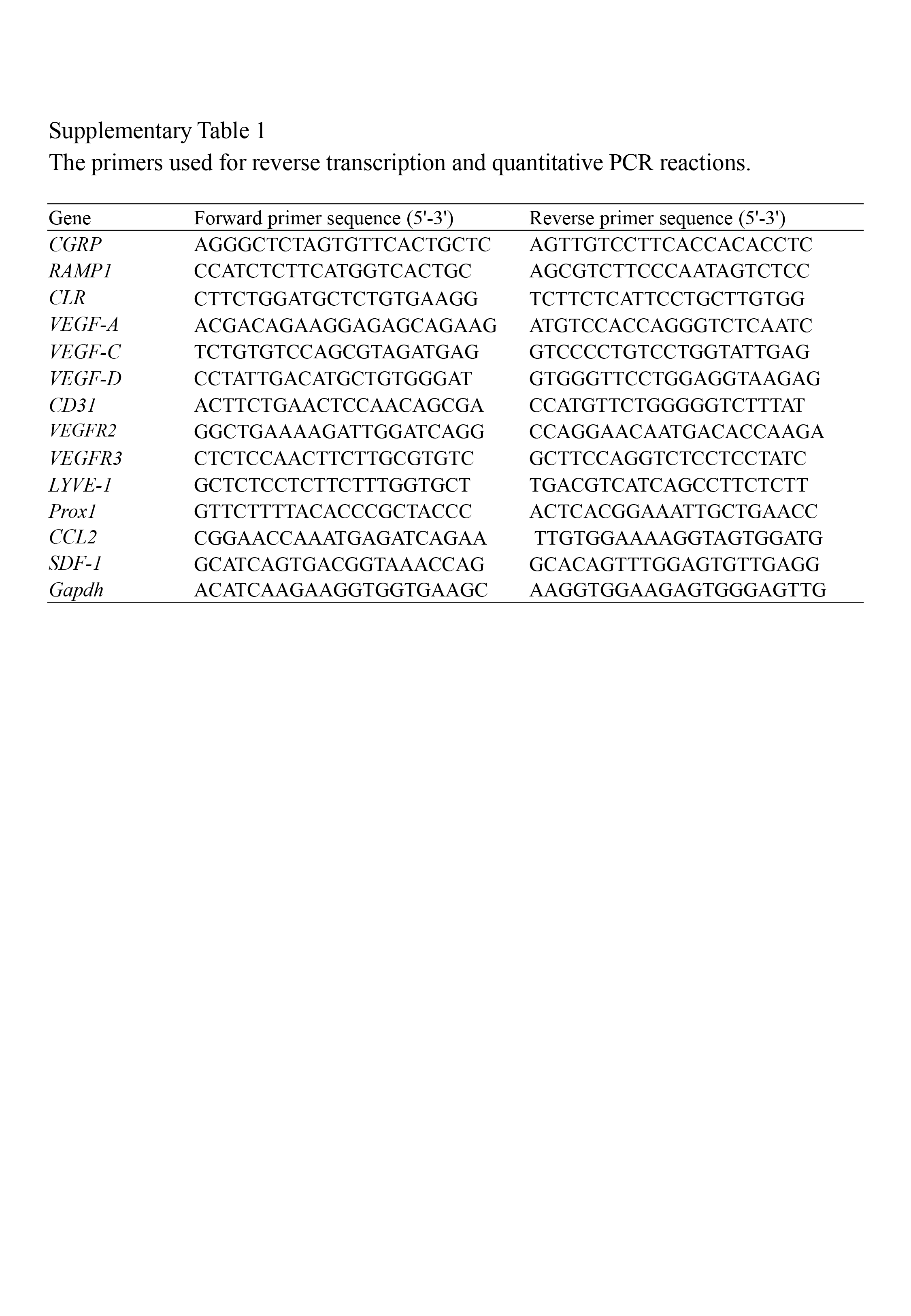

Supplement: Supplementary file 3 — Table S1 [file JCMM-24-11984-s003.tif]
