# Supplementary material for: Inhibition of receptor activity–modifying protein 1 suppresses the development of endometriosis and the formation of blood and lymphatic vessels
Source: J Cell Mol Med. 2020 Sep 1;24(20):11984–97. doi: 10.1111/jcmm.15823 (PMC7578853; doi:10.1111/jcmm.15823)
Supplement: Supplementary file 4 — Supplementary Material [file JCMM-24-11984-s004.docx]

**Supplementary Figure 1.** **Growth of and angiogenesis/lymphangiogenesis within endometrial tissue implants during the development of endometriosis in WT→WT mice.**

(A) Temporal changes in implant area after WT→WT transplantation. Data are expressed as the mean ± SD (n = 5 mice per time-point). *p < 0.05. (Right-hand panels) Typical appearance over time of implants in the WT→WT after endometrial transplantation. Scale bars, 2 mm.

(B) (Left-hand panel) Double immunofluorescence staining of CD31 (red) and LYVE-1 (green) in whole mount endometrial tissues from WT→WT mice on Day 14. Scale bar, 200 μm.

(Middle panel) Immunohistochemical staining of CD31 in endometrial tissue implant sections from WT→WT mice on Day 14. Scale bar, 200 μm. Arrows indicate CD31-positive cells.

(Right-hand panel) Immunohistochemical staining of LYVE-1 in endometrial tissue implant sections from WT→WT mice on Day 14. Scale bar, 200 μm. Arrows indicate LYVE-1-positive cells.

(C) Time course of changes in MVD of the endometrial tissue implants in the WT→WT mice. Data are expressed as the mean ± SD (n = 4 mice per time-point). *p < 0.05. (Right-hand panels) Immunohistochemical staining of CD31 in endometrial tissue implant sections from WT→WT mice on Day 14. Scale bar, 50 μm. The arrow heads indicate CD31^+^ cells.

(D) Time course of changes in LVD of the endometrial tissue implants in the WT→WT mice. Data are expressed as the mean ± SD (n = 3-5 mice per time-point). *p < 0.05. (Right panels) Immunohistochemical staining of LYVE-1 in endometrial tissue implant sections from WT→WT mice on Day 14. Scale bar, 50 μm. Arrow heads indicate LYVE-1^+^ cells.

(E) Expression levels of mRNA encoding RAMP1 and CLR over time in endometrial tissue implants from WT→WT mice. Data are expressed as the mean ± SD (n = 3–6 mice per group). *p < 0.05.

**Supplementary Figure 2. RAMP1 expression in the implant tissue.**

Immunofluorescence staining of RAMP1 (red) in the endometrial tissue implants from WT→WT mice on Day 14. The dotted line indicates the border between the peritoneum and implant tissue. Scale bars, 100 μm.
